# Frailty and GLIM-defined malnutrition contribute to poor clinical outcomes in older adult inpatients in the general surgery department

**DOI:** 10.3389/fnut.2025.1435429

**Published:** 2025-06-30

**Authors:** Chengyu Liu, Liru Chen, Peng Liu, Lei Li, Bo Cheng, Jingyong Xu, Hongyuan Cui, Mingwei Zhu

**Affiliations:** ^1^Department of General Surgery, Department of Hepato-bilio-pancreatic Surgery, Beijing Hospital, National Center of Gerontology, Institute of Geriatric Medicine, Chinese Academy of Medical Sciences, Beijing, China; ^2^Department of Nutrition, Beijing Hospital, National Center of Gerontology, Institute of Geriatric Medicine, Chinese Academy of Medical Sciences, Beijing, China

**Keywords:** frailty, malnutrition, aged, general surgery, clinical outcome

## Abstract

**Background and aims:**

Frailty and malnutrition are prevalent among older adult inpatients. Our study aimed to analyze the correlation between frailty and malnutrition and determine their effects on the clinical outcomes in older adult surgical inpatients.

**Methods:**

This cross-sectional observational study included older adult inpatients (≥ 65 years old) undergoing scheduled surgery. Anthropometric measurements and hematological examination results were collected at the time of admission. Frailty and malnutrition were assessed using the frailty phenotype and the Global Leadership Initiative on Malnutrition (GLIM) criteria. Nutritional support during hospitalization and clinical outcomes, such as the occurrence of postoperative complications, in-hospital death, length of hospital stays, and hospital costs, were recorded. The chi-squared and rank-sum tests were used for comparison. Univariate and multivariate logistic regression analyses were used to calculate the odds ratios (OR) and 95% confidence intervals (CI) for frailty, malnutrition, and postoperative complications.

**Results:**

In 394 patients, the frailty prevalence was 17.3% (68/394), and 146 inpatients (37.1%) were malnourished. The overlapping prevalence rate of frailty and malnutrition was 12.2% (48/394). Frailty and malnutrition were correlated (r = 0.464, *p* < 0.001). Multivariate analysis revealed that frailty significantly increased the risk of postoperative complications (OR: 2.937, 95% CI: 1.475–5.850, *p* = 0.002). There were significant differences in the length of hospital stays and hospital costs among the four groups of patients with frailty and malnutrition, frailty and no malnutrition, malnutrition and no frailty, and no frailty and malnutrition (*p* < 0.001; *p* < 0.001).

**Conclusion:**

A significant positive correlation was observed between frailty and malnutrition. Frailty and malnutrition are significantly associated with adverse clinical outcomes. Therefore, it is necessary to manage frailty and malnutrition to improve the prognosis.

## 1 Background

Frailty is an age-related clinical syndrome characterized by a decline in the physiological capabilities of multiple organ systems, resulting in increased vulnerability to stressful events (1). Malnutrition is a state of altered body composition (loss of fat mass) and decreased body cell mass due to insufficient intake or absorption of nutrients, leading to decreased physical and mental function and impaired clinical outcomes of disease (2). Malnutrition is caused by diseases, hunger, improper diet, poor physical function, poor appetite, and psychological problems (3, 4). The Global Leadership Initiative on Malnutrition (GLIM) proposed a global consensus on the diagnosis of malnutrition in adults in clinical settings in 2019 (3). The first step involves identifying “at risk” individuals to use validated screening tools, and the second step involves assessing the diagnosis and severity of malnutrition, with one phenotypic and one etiologic criterion to diagnose malnutrition (3). Frailty and malnutrition are widespread among older hospitalized patients and are common problems in geriatric surgical patients (5, 6). With the rapid increase in population aging, the prevalence of frailty in older adult patients has increased, which puts greater pressure on global healthcare systems (1).

These recent cross-sectional studies reveal that frailty and malnutrition are closely related (7, 8). A meta-analysis that pooled 10 studies (2,427 patients) shows that there is considerable overlap (49.7%) between physical (pre-)frailty and (risk of) malnutrition in hospitalized older adults (5). Evidence-based studies have demonstrated that frailty and malnutrition, even independently, can sufficiently contribute to adverse clinical outcomes. Frailty is associated with a higher risk of death, functional dependence, longer length of hospital stays (LOS), and impaired quality of life (9–11). In addition, malnutrition is associated with poor clinical outcomes, including increased incidence of complications, mortality, longer LOS, and higher hospital costs (12, 13). There is a lack of analysis combining the Global Leadership Initiative on Malnutrition (GLIM) criteria with other acceptable diagnostic methods of frailty in older adult surgical patients to analyze their impact on clinical outcomes (14).

The purpose of our study was to investigate the prevalence of frailty and malnutrition among older adult inpatients in the Department of General Surgery, analyze the correlation between frailty and malnutrition, and determine their impact on clinical outcomes, including the incidence of postoperative complications.

## 2 Methods

### 2.1 Study design and subjects

This is a single-center cross-sectional observational study conducted from 1 January 2021 to 1 July 2022. Older adult inpatients undergoing scheduled surgery were recruited from the Department of General Surgery of Beijing Hospital. Three investigators conducted the study and received centralized training on research methods before the study began. A case report form was filled out at the time of admission. Baseline surveys, frailty assessments, and nutritional assessments were conducted, and outcome information was collected after discharge. All collected data were then entered into an Excel spreadsheet.

Prior to the investigation, the study was approved by the Ethics Committee of Beijing Hospital (approval number: 2020BJYYEC-265-01) and registered in the Chinese Clinical Trial Registry (registration number: ChiCTR2000040512) on 1 December 2020. Informed consent was obtained from all patients.

The inclusion criteria for the participants were as follows: (1) age 65 years or older; (2) undergoing general surgical treatment; (3) being conscious; and (4) being willing to accept assessment and provide signed informed consent. The exclusion criteria for the participants were as follows: (1) inability to cooperate with the evaluation procedure (such as severe cognitive impairment, communication difficulties, and critical illness); (2) refusal to sign the informed consent form; (3) withdrawal of informed consent; and (4) refusal to undergo evaluation.

### 2.2 Data collection

Demographic information such as gender, age, and marital status was recorded. Anthropometric measurements were taken after admission to record the height, weight, right upper arm circumference, right calf circumference, and right handgrip strength. Hematological examination, including lymphocyte count, serum albumin, and serum total protein, was conducted after admission. Serum albumin levels can serve as a supportive proxy measure of inflammation, and a concentration of < 35 g/L was defined as hypoalbuminemia (15, 16). Diagnoses of malignant tumors, biliary calculi, and other diseases were collected from the home pages of inpatients and discharge medical records. Comorbidities such as diabetes mellitus, hypertension, coronary heart disease, and a history of cerebral infarction were recorded. Nutritional support was defined as follows: parenteral nutrition (PN) was defined as the infusion of intravenous macronutrients to provide energy of 10 kcal/kg/day for at least 3 days; enteral nutrition (EN) was defined as the continuous use of oral nutritional supplements (> 500 kcal/day) or gavage (> 10 kcal/kg/day) for at least 3 days; and PN + EN was defined as the use of both parenteral and enteral nutrition, with an energy supply of not less than 80% of the target intake. Otherwise, the patients were considered as not receiving any nutritional support (17).

The primary outcome was postoperative complications, defined according to the Clavien–Dindo classification system grades II-V (18). Secondary outcomes included all-cause in-hospital death, LOS, and total hospital costs. The length of stay (LOS) was defined as the cumulative number of days from admission to discharge. Hospital costs refer to the medical expenses incurred by hospitalization due to illness, which are determined according to the collection vouchers of medical expenses and hospitalization expenses issued by medical institutions, combined with medical records, diagnosis reports, and other relevant evidence.

### 2.3 Fried frailty phenotype

The frailty phenotype method is recommended for frailty assessment according to the Frailty Guidelines of the International Conference of Frailty and Sarcopenia Research (19).

The frailty phenotype identifies five characteristics of frailty, namely, weight loss, slow walking speed, decreased handgrip strength, low physical activity, and fatigue. Those with three or more indicators were diagnosed as frail, those with one or two indicators as pre-frail, and those without the above five indicators as robust (20). Pre-frail and robust patients were collectively referred to as non-frail patients.

### 2.4 Nutritional screening and assessment

The prevalence of nutritional risk and malnutrition was prospectively defined using the Nutritional Risk Screening 2002 (NRS-2002) and the GLIM criteria. The NRS-2002 included three aspects: impaired nutritional status, disease severity, and age ≥ 70 years. A total score of 3 or higher indicates that a patient is at nutritional risk (21). Patients identified as being at nutritional risk were further assessed for malnutrition diagnosis and severity using the GLIM criteria. The GLIM criteria include three phenotypic components (non-volitional weight loss, low BMI, and reduced muscle mass) and two etiologic components (reduced food intake or assimilation, and disease burden or inflammation). A diagnosis of malnutrition requires the presence of at least one phenotypic criterion and one etiological criterion (3).

In the phenotypic criteria, weight loss > 5% within the past 6 months or >10% beyond 6 months was considered to be non-volitional weight loss; we evaluated low BMI using Asian BMI data (18.5 kg/m^2^ and 20 kg/m^2^ for patients aged < 70 years and ≥ 70 years, respectively). Appendicular skeletal muscle was measured using direct segmental multi-frequency bioelectrical impedance analysis with InBody 720 device. Reduced muscle mass was defined by the appendicular skeletal muscle index (ASMI), with cutoff values of < 7 kg/m^2^ for men or < 5 kg/m^2^ for women. For the etiologic criteria, reduced food intake or assimilation was defined as energy intake of less than 50% of the required amount over a period of 1 week, or any decrease in energy intake sustained over 2 weeks. Disease burden or inflammation was considered present in patients with either an acute illness or injury (e.g., a serious infection) or a chronic disease (e.g., a malignant disease). Severe malnutrition was diagnosed when either of the following conditions was met: (i) weight loss > 10% within the past 6 months or > 20% beyond 6 months; (ii) BMI < 17.0 kg/m^2^ for individuals < 70 years or < 17.8 kg/m^2^ for those aged 70 years or older. Moderate malnutrition was defined as the diagnosis of malnutrition without meeting severe malnutrition conditions.

### 2.5 Sample size

This study used a cross-sectional observational design. The exposure group was the frail group, and the control group was the non-frail group. The incidence of postoperative complications was the observed outcome indicator. According to a previous meta-analysis (22), the incidence of frailty was expected to be 20%, so the sample size ratio between the exposed and control groups was prespecified as 1:4. The incidence of complications was predicted to be 24% in the experimental group and 5% in the control group (22). With a two-sided *α* = 0.05 and a power of 80%, PASS 15 software was used to calculate the sample size of the exposure group. The minimum sample size of the exposure group was 50 and that of the control group was 200. Accounting for an estimated 10% of participants potentially lost to follow-up or refusing follow-up, a total sample size of more than 278 participants was determined for inclusion.

### 2.6 Statistical analysis

SPSS software (Version 25.0. Armonk, NY: IBM Corp.) was used for data analysis. The Shapiro–Wilk test was used to determine the distribution type of the quantitative variables, and the homogeneity of variance was evaluated using the *F*-test. Normally distributed variables were expressed as means (standard deviation), non-normally distributed variables were expressed as medians (interquartile range), and categorical variables were expressed as numbers (percentages). The chi-squared test was used for differences in the distribution of categorical variables between the groups. For continuous variables, analysis of variance (ANOVA), the Mann–Whitney rank-sum test, or the Kruskal–Wallis rank-sum test were used for comparison between different groups. Spearman’s correlation was used to analyze the correlation between frailty and malnutrition, and the chi-squared test was used to analyze the correlation between five indicators of the frailty phenotype diagnosis and malnutrition. Logistic regression analysis was conducted to explore independent risk factors for postoperative complications. A univariate logistic regression (input) analysis was conducted to examine the correlation between risk factors and postoperative complications, and for categorical variables that are statistically significant in the univariate logistic regression analysis, along with identified confounding factors such as age and gender, a multivariate logistic regression analysis using the forward: likelihood ratio was conducted. An interaction analysis between frailty and malnutrition was performed using an ANOVA test for LOS and hospital costs. A two-tailed *p-*value of <0.05 was considered as statistically significant.

## 3 Results

### 3.1 Patient characteristics

Among the 394 older adult inpatients enrolled, with a median age of 71 (interquartile range of 8) years, 134 (34.0%) had biliary calculi, 74 (18.8%) had colorectal cancer, 28 (7.1%) had gastric cancer, 60 (15.2%) had other gastrointestinal malignancies, and 98 (24.9%) had other diseases. There were 199 (50.5%) men and 195 (49.5%) women. The baseline characteristics of the enrolled inpatients are shown in Table 1.

**Table 1:** Demographic and clinical characteristics of older adult inpatients with frailty and malnutrition in the Department of General Surgery.

Characteristics	Frailty			Nutritional status		
	Frailty (*n* = 68)	Without frailty (*n* = 326)	*p*-value	Malnutrition (*n* = 146)	Without malnutrition (*n* = 248)	*p*-value
Age (years)	74.5 (13.0)	70.0 (7.0)	<0.001	72.0 (10.0)	70.0 (7.0)	<0.001
Sex, female (%)	32 (47.1%)	163 (50.0%)	0.659	68 (46.6%)	127 (51.2%)	0.374
Height (cm)	163.4 (12.7)	163.3 (12.4)	0.773	163.3 (13.1)	163.3 (11.5)	0. 768
Weight (kg)	62.0 (12.9)	65.0 (12.7)	0.026	61.0 (12.4)	66.1 (13.4)	<0.001
BMI (kg/m^2^)	22.9 (4.4)	24.2 (3.9)	0.010	22.8 (3.7)	24.6 (4.4)	<0.001
CC (cm)	33.5 (4.4)	34.3 (4.5)	0.026	33.5 (4.5)	34.5 (4.5)	0.003
Upper arm circumference (cm)	26.1 (4.2)	26.8 (4.0)	0.215	26.0 (3.7)	27.0 (3.5)	< 0.001
Handgrip strength (kg)	19.35 (10.3)	25.15 (11.7)	<0.001	22.1 (10.7)	25.4 (12.3)	0.001
TLC (10^9^/ L)	1.77 (0.95)	1.80 (0.92)	0.956	1.79 (1.10)	1.79 (0.90)	0.572
Serum albumin (g/dL)	3.65 (0.70)	3.90 (0.40)	<0.001	3.70 (0.60)	3.90 (0.30)	0.015
Serum total protein (g/dL)	6.45 (0.60)	6.50 (0.70)	0.036	6.40 (0.80)	6.50 (0.60)	0.073
Appendicular skeletal muscle (kg)	16.76 (4.39)	16.56 (4.85)	0.720	16.55 (4.78)	16.82 (6.49)	0.216
Skeletal muscle (kg)	22.65 (4.70)	22.65 (5.20)	0.258	22.65 (5.03)	22.90 (5.10)	0.160
Body fat (kg)	17.70 (10.73)	19.7 (9.45)	0.142	18.35 (10.17)	19.90 (9.27)	0.017
Fat-free mass (kg)	42.60 (8.48)	42.35 (9.68)	0.482	42.10 (9.20)	42.95 (10.45)	0.497

All values are expressed as median (IQR) or *n* (%). CC, calf circumference; TLC, total lymphocyte count; IQR, interquartile range.

### 3.2 Correlation analysis between frailty and malnutrition

In total, 68 inpatients (17.3%) were frail, 201 (51.0%) were pre-frail, and 125 (31.7%) were robust.

The incidence of nutritional risk was 48.2% (190/394). A total of 146 inpatients (37.1%) had malnutrition, including 88 (22.3%) with moderate malnutrition and 58 (14.7%) with severe malnutrition.

The overlap between frailty and malnutrition was 12.2% (48/394), and the overlap between pre-frailty and malnutrition was 21.8% (86/394). A total of 20 patients (5.1%) had frailty but no malnutrition, 98 (24.9%) had malnutrition but no frailty, and 228 patients (57.9%) had neither frailty nor malnutrition.

Frailty was positively correlated with malnutrition, and the Spearman correlation coefficient r for frailty and malnutrition was 0.464 (*p* < 0. 001) (Table 2). All five diagnostic indicators of the frailty phenotype were significantly associated with the occurrence of malnutrition (all *p* < 0.05) (Table 3).

**Table 2:** Overlapping prevalence of frailty and malnutrition [*n* (%)].

Frailty phenotype	GLIM criteria		
	Without malnutrition	Moderate malnutrition	Severe malnutrition
Robust	113 (28.7%)	11 (2.8%)	1 (0.3%)
Pre-frailty	115 (29.2%)	58 (14.7%)	28 (7.1%)
Frailty	20 (5.1%)	19 (4.8%)	29 (7.4%)

**Table 3:** Correlation between diagnostic indicators of the frailty phenotype and malnutrition in older adult inpatients in the Department of General Surgery [n (%)].

Frailty phenotype indicators	With malnutrition (*n* = 146)	Without malnutrition (*n* = 248)	*χ* ^2^	*p*-value
Weight loss			172.984	< 0.001
Yes	105 (71.9%)	20 (8.1%)		
No	41 (28.1%)	228 (91.9%)		
Slow gait speed Slowness			20.032	< 0.001
Yes	46 (31.5%)	32 (12.9%)		
No	100 (68.5%)	216 (87.1%)		
Weakness			17.452	< 0.001
Yes	77 (52.7%)	78 (31.5%)		
No	69 (47.3%)	170 (68.5%)		
Low physical activity			6.120	0.013
Yes	51 (34.9%)	58 (23.4%)		
No	95 (65.1%)	190 (76.6%)		
Exhaustion			4.303	0.038
Yes	26 (17.8%)	26 (10.5%)		
No	120 (82.2%)	222 (89.5%)		

### 3.3 Univariate and multivariate analyses of postoperative complications

The overall postoperative complication rate was 13.5% (53/394). Frailty (*p* = 0.001), nutritional risk (*p* < 0.001), malnutrition (*p* < 0.001), age (*p* = 0.018), hypoalbuminemia (*p* = 0.022), coronary heart disease (*p* = 0.001), malignant tumor (*p* < 0.001), PN (*p* < 0.001), and PN + EN (*p* = 0.003) were significantly associated with the incidence of postoperative complications. However, sex (*p* = 0.068), marital status (*p* = 0.379), medical expenses type (*p* = 0.270), education level (*p* = 0.406), BMI (*p* = 0.054), diabetes mellitus (*p* = 0.538), hypertension (*p* = 0.703), a history of cerebral infarction (*p* = 0.659), and EN (*p* = 0.069) were not significantly associated with the incidence of postoperative complications (Table 4).

**Table 4:** Univariate and multivariate regression analyses of postoperative complications.

Characteristics	Category	OR	Univariate regression analysis		OR	Multivariate regression analysis	
			95% CI	*p*-value		95% CI	*p*-value
Sex	Female	0.575	0.317–1.042	0.068			0.203
	Male	Reference					
Age^a^		1.058	1.010–1.109	0.018			0. 443
Marital status	Married	1.342	0.697–2.584	0.379			
	Widowed or other	Reference					
Medical expenses type	Other	1.583	0.809–3.100	0.180			
	Medical insurance	Reference					
Education level	College or above	0.768	0.399–1.477	0.428			
	Middle school	1.667	0.754–3.682	0.207			
	Primary school and below	Reference					
BMI^a^		0.922	0.848-1.001	0.054			
Hypoalbuminemia	Yes	2.275	1.125–4.601	0.022			0.250
	No	Reference					
Diabetes mellitus	Yes	1.231	0.635–2.384	0.538			
	No	Reference					
Hypertension	Yes	0.893	0.500–1.597	0.703			
	No	Reference					
Coronary heart disease	Yes	3.148	1.585–6.252	0.001	2.615	1.255–5.449	0.010
	No	Reference					
Malignant tumor	Yes	4.804	2.392–9.649	<0.001	4.713	2.307–5.850	<0.001
	No	Reference					
History of cerebral infarction	Yes	1.317	0.481–3.604	0.592			
	No	Reference					
Frailty	Yes	2.993	1.574–5.692	0.001	2.937	1.475–5.850	0.002
	No	Reference					
Nutritional risk	Yes	4.403	2.235–8.672	<0.001			
	No	Reference					
Malnutrition	Yes	3.034	1.674–5.499	<0.001			0.093
	No	Reference					
Frailty × Malnutrition	Yes	2.841	1.387–5.819	0.004			0.709
	No	Reference					
Nutritional support	PN	0.038	0.016–0.090	<0.001			
	EN	0.431	0.174–1.067	0. 069			
	PN + EN	0.246	0.097–0.627	0.003			
	No	Reference					

BMI, body mass index; OR, odds ratio; CI, confidence interval; PN, parenteral nutrition; EN, enteral nutrition.

a Indicated as quantitative variables.

After excluding the confounding factors such as gender, age, hypoalbuminemia, coronary heart disease, and malignant tumor, frailty was an independent risk factor for postoperative complications [odds ratio (OR): 2.937, 95% confidence intervals (CI): 1.475–5.850, *p* = 0.002]. In addition, there was no multiplicative interaction between frailty and malnutrition (*p* = 0.709) (Table 4).

### 3.4 Effects of frailty and malnutrition on LOS and hospital costs

Significant differences in the LOS were observed among the four groups of patients with frailty and malnutrition, frailty and no malnutrition, malnutrition and no frailty, and no frailty and malnutrition, with mean (SD) values of 15 (12), 9 (12), 15 (15), 9 (10) days, respectively (*p* < 0.001). The interaction of frailty and malnutrition between the two factors had no significant effect on the LOS (*F* = 0.279, *p* = 0.757).

Significant differences in hospital costs were observed among the four groups, with mean (SD) values of 8.08 (12.54), 2.69 (3.39), 10.79 (11.63), 2.65 (7.56) thousand US dollars, respectively (*p* < 0.001). However, the interaction between the two factors did not have a significant effect on hospital costs (*F* = 0.655, *p* = 0.520).

### 3.5 Nutritional support during hospitalization

The total proportion of inpatients who received nutritional support was 33.5% (132/394), including 8.9% (35/394) receiving PN, 10.9% (43/394) receiving EN, and 13.7% (54/394) receiving PN + EN.

Among inpatients with malnutrition, 50% (73/146) received nutritional support, while 23.8% (59/248) of inpatients without malnutrition received nutritional support. There was a significant difference in nutritional support between the two groups (*χ*^2^ = 28.337, *p* < 0.001).

## 4 Discussion

This study found a significant positive correlation between frailty and malnutrition. Frailty is an independent risk factor for postoperative complications, and patients with both frailty and malnutrition tend to have poor clinical outcomes. The association between frailty and malnutrition is reflected by the fact that they share common pathophysiological pathways, including body tissue loss and chronic inflammation. Symptoms such as weight loss, slow gait speed, decreased handgrip strength, and fatigue are characteristic of both malnutrition and frailty (23–26). Additionally, frailty and malnutrition are associated with decreased intrinsic capacity and share common sociodemographic, physical, and cognitive risk factors (27). Low intake of energy, protein, and vitamin D is associated with the onset and progression of frailty and malnutrition. Patients with frailty often have insufficient food intake, making it difficult to meet their nutritional requirements, including energy and protein. Patients in the pre-frailty stage or frailty stage typically exhibit poor nutritional status (28). Malnutrition and frailty are intricately connected in their pathogenesis, influencing each other in a complex and synergistic manner (29).

Screening assessment tools for frailty and malnutrition also have overlapping factors such as weight loss and impaired physical function (3, 6, 20). One factor of the frailty phenotype is decreased handgrip strength, which is a manifestation of reduced muscle mass in the diagnosis of malnutrition, and low physical activity is consistent with mobility in the subjective global assessment for malnutrition (3, 30). This is supported by our finding that all five of the frailty phenotypes were associated with the diagnosis of malnutrition. A prospective study by Khajoueinejad et al. (31) found that, in combination with preoperative nutritional indicators, the frailty assessment tool risk analysis index can be used to identify patients with abdominal malignancies who may benefit from additional preoperative risk stratification and increased postoperative evaluation. Emerging approaches that combine nutritional assessment and frailty diagnosis can objectively identify patients at risk during the perioperative period and guide perioperative treatment (6).

Active aging and regular physical activity can significantly improve older adults’ physical function, cognitive ability, and mental health, thereby delaying the progression of frailty and enhancing quality of life (32, 33). However, age-related physiological changes may exacerbate frailty risk, while sedentary behavior and insufficient physical activity can further accelerate this process (34). Cocnurrently, malnutrition is a key contributing factor to frailty, with relevant mechanisms including insufficient nutrient intake, increased metabolic demands, and reduced nutrient bioavailability (35, 36). These findings underscore the need for comprehensive interventions targeting both physical activity and nutritional status to mitigate frailty and its adverse outcomes.

In our study, the multivariate analysis revealed that frailty was an independent risk factor for the occurrence of postoperative complications, whereas malnutrition had no significant effect, and there was no multiplicative interaction between them. Possible reasons include the following: the frailty assessment already encompasses comprehensive risks such as nutritional deficiencies, thereby diminishing the independent contribution of malnutrition; the regression analysis included only 53 complication cases, potentially resulting in insufficient statistical power; and the mechanisms of postoperative complications are complex—while malnutrition may exert indirect effects (e.g., delaying recovery), frailty has a more direct and significant impact on physiological reserve. However, large-scale studies support the association between frailty, malnutrition, and adverse clinical outcomes, suggesting that they may have additive rather than synergistic effects. Tjeertes et al. (37) included 56 studies with a total of 1, 106, 653 patients in a meta-analysis and found that frailty significantly increased the risk of 30-day mortality in older adult patients undergoing non-cardiac surgery. Specifically, frailty was associated with a higher risk of 30-day mortality (31 studies, 673,387 patients; relative risk: 3.71, 95% CI: 2.89–4.77) and 30-day complications (37 studies, 627,991 patients; relative risk: 2.39, 95% CI: 2.02–2.83), and the risk of 1-year mortality was increased by a factor of 3 (6 studies, 341,769 patients; relative risk: 3.40, 95% CI: 2.42–4.77). A recent meta-analysis by Matsui et al. (38) found that malnutrition may worsen overall survival (hazard ratio: 1.56; 95% CI: 1.38–1.75) and increase the risk of postoperative complications (relative risk: 1.82; 95% CI: 1.28–2.60). Chew et al. (39) found that, the more severe the frailty, the more likely the patients were to expereince geriatric syndromes such as cognitive impairment, falls, and malnutrition. Frail patients with geriatric syndromes also had longer LOS and increased 30-day readmission rates. Stretton et al. (40) conducted a large multicenter cohort study involving 21,976 surgical patients, which showed that, after controlling for confounding factors, malnutrition, and socioeconomic status, the average LOS for patients at high risk of frailty was 3.46 times longer (mean ratio: 3.46; 95% CI: 3.20–3.73). In addition, poor nutritional status was significantly associated with worse clinical outcomes (40).

Frailty clinical practice guidelines strongly recommend multicomponent exercise programs, including resistance training, for the management of frailty. Protein and calorie supplementation is also recommended when weight loss or malnutrition occurs (19). Recent trials support that nutritional supplementation combined with exercise training may be more effective in frailty interventions (41). A systematic review found that frailty is associated with a low intake of micronutrients and protein, while a high dietary antioxidant capacity is associated with a lower risk of developing frailty (28). A longitudinal study by Rabassa et al. (42) found that habitual antioxidant diet in older adults was associated with a lower risk of frailty syndrome, suggesting that antioxidant dietary interventions could help manage frailty.

Frailty and malnutrition are significantly associated with adverse clinical outcomes. The principles of individualized medicine should guide in the management of malnutrition and frailty in older adult surgical patients, and the benefits of interventions should not outweigh the potential harm to patients (19). Malnourished or frail older adult surgical patients are more likely to undergo unnecessary tests and treatments, which may expose them to unnecessary burdens and risks. These findings support the use of GLIM criteria and frailty phenotypes to assess malnutrition and frailty in older adult general surgical inpatients for the intervention and improvement of clinical outcomes and may help general surgeons select appropriate preoperative risk stratification assessment tools to help determine the best treatment strategy. Future clinical studies could explore the benefits of personalized interventions in frail and malnourished older adult patients undergoing surgery.

Our study has two strengths. The frailty phenotype and GLIM criteria, which are globally recognized as standard diagnostic methods, were used to investigate the prevalence of frailty and malnutrition among older adult general surgery inpatients. In addition, we comprehensively analyzed the impact of frailty and malnutrition on clinical outcomes, such as postoperative complications, in-hospital death, LOS, and hospital costs.

Our study has some limitations. First, it was a single-center, small-sample, cross-sectional observational study; future studies should involve several research institutions and a large sample size. Second, surgery-related risk factors, such as preoperative American Society of Anesthesiologists classification, operation time, and intraoperative blood loss, were not examined, and subgroup analyses based on surgical methods were not performed owing to the small sample size and the insufficient effective sample size for regression analysis. Third, frailty and nutritional status are dynamic and may vary significantly depending on the frailty and nutritional status of patients with different disease states and treatment stages. Future studies should dynamically investigate frailty and malnutrition during admission and discharge.

In conclusion, there was a significant positive correlation between frailty and malnutrition among older adult inpatients in the General Surgery department. Frailty and malnutrition are significantly associated with adverse clinical outcomes.

## Data Availability

The raw data supporting the conclusions of this article will be made available by the authors, without undue reservation.
